# Development of surfactant formulation for high-temperature off-shore carbonate reservoirs

**DOI:** 10.3389/fchem.2024.1408115

**Published:** 2024-08-07

**Authors:** Krishna Panthi, Kishore K. Mohanty

**Affiliations:** Hildebrand Department of Petroleum and Geosystems Engineering, The University of Texas at Austin, Austin, TX, United States

**Keywords:** surfactant, SP flood, high temperature, enhanced oil recovery, carbonate reservoirs

## Abstract

The residual oil left behind after water flooding in petroleum reservoirs can be mobilized by surfactant formulations that yield ultralow interfacial tension (IFT) with oil. However, finding ultralow IFT surfactant formulations is difficult for high-temperature, off-shore, carbonate reservoirs. These reservoirs are often water-flooded with seawater (with a lot of divalent ions), which is often incompatible with many surfactants at high temperatures. The goal of this research is to develop a surfactant formulation for an off-shore carbonate reservoir at 100°C previously flooded by seawater. Surfactant–oil–brine phase behavior was studied for formulations, starting from a single surfactant to mixtures of surfactants and a co-solvent. Mixtures of three surfactants and one co-solvent were needed to produce ultralow IFT formulations for the oil of interest. The surfactant system with polymer mobility control was tested in crushed reservoir rock packs. The cumulative oil recovery was >99% for the surfactant–polymer (SP) flood with an optimal salinity gradient. The constant salinity SP floods with seawater increased oil recovery significantly beyond the water flood (cumulative oil recovery >91%), even though the recovery was lower than that of the optimal salinity gradient SP flood. Our experimental work demonstrates the effectiveness of the surfactant formulation for a high-temperature carbonate reservoir at seawater salinity.

## 1 Introduction

Approximately 60% of the worldwide oil reserves are carbonates (Sheng, 2013a). Carbonate reservoirs tend to be oil-wet/mixed-wet and heterogeneous at many scales. Some of the reservoirs are deep, and the temperature is high (∼100°C). Primary and secondary recovery techniques can recover only approximately one-third of the original oil in place (OOIP). Enhanced oil recovery (EOR) techniques are needed to recover the oil left behind in the reservoirs. Surfactant-enhanced oil recovery is one of the techniques that mobilizes trapped oil by lowering the interfacial tension (IFT) between water and oil (Hirasaki et al., 2011). This technology is mature at lower temperatures and for sandstones. However, surfactant flooding for high-temperature, high-salinity (HTHS) carbonates is still challenging. Off-shore carbonate reservoirs are often water-flooded with seawater (with a lot of divalent ions), being incompatible with many surfactants at high temperatures. Anionic surfactants tend to adsorb on positively charged carbonate rocks. Heterogeneity and oil-wetness lead to bypassing of low-permeability regions at multiple scales during floods.

Alkaline surfactant polymer (ASP) flooding has been implemented in oilfields over the last 25 years, and they have been proven effective (Olajire, 2014; Zhu, 2015). The ASP involves the alkalis, polymers, surfactants, and sometimes co-solvents in the application process (Lake, 1989; Green and Willhite, 1998). The ASP process mobilizes the residual oil by reducing the capillary forces in the reservoir rock (Stegemeier, 1977; Chatzis et al., 1983; Pope et al., 2000), and the polymers are used for mobility control (Lake, 1989; Green and Willhite, 1998). Typically, surfactants are used in chemical EOR to reduce the interfacial tension (IFT) between oil and water phases (Panthi et al., 2020). Alkali helps increase pH, which reduces surfactant retention, and can react with acidic oil components to form soap (Nelson et al., 1984; Solairaj et al., 2012; Sharma et al., 2015; Southwick et al., 2018; Wang et al., 2018). However, divalent cations present in the connate water can precipitate with the injected alkali and cause scaling build-up near the well bore and the inner surface of the transferring pipes (Jiecheng et al., 2008; Goswami et al., 2018). Alkali also enhances corrosion, which increases the operational issues and maintenance cost (Gang et al., 2007; Jiecheng et al., 2011). ASP processes need an injection of brine with little or no divalent cations.

Many carbonate reservoirs often contain high-salinity and high-hardness brine, especially high-temperature reservoirs. Off-shore reservoirs are often flooded with seawater which contains high hardness, and it is the easily available injection water for chemical floods. ASP floods cannot be applied for such reservoirs because of the hardness in the injection brine; thus, surfactant–polymer (SP) floods are considered. However, interaction with seawater can lead to precipitation or degradation of surfactants and polymers (Kamal et al., 2018; Puerto et al., 2018). A substantial amount of calcium and magnesium ions can make the commonly used anionic surfactants and polymers less effective and can result in higher surfactant adsorption (Levitt et al., 2011; Dupuis et al., 2017a). SP formulations in lab tests were reported for high-salinity, high-hardness reservoirs (Tabary et al., 2013) below 85°C. A single-well SP pilot test was also conducted in an off-shore UAE well at 83°C, but there was a problem with polymer plugging (Levitt et al., 2011).

At HTHS conditions, many challenges have been encountered in preparing SP formulations (Kalfoglou, 1977; Wu et al., 2007; Wang et al., 2008). It is difficult to find surfactants that have aqueous solubility at HTHS conditions (Kamal et al., 2018), and only a few formulations have been identified for low IFT. For example, Puerto et al. (2018) developed a surfactant formulation for imbibition into fractured dolomites at 100°C, which involved three surfactants, and the adsorption was approximately 0.5 mg/g of rock. Janjua et al. (2018) used a mixture of a viscoelastic surfactant and a chelating agent to develop a viscous fluid to recover viscous oil from HTHS carbonate reservoirs. Cai et al. (2019) developed formulations with a Guerbet alkoxy betaine surfactant stable in HTHS brine, and it gives low IFT. Panthi et al. (2022) developed a surfactant formulation for an onshore HTHS limestone reservoir. The commonly used hydrolyzed polyacrylamide (HPAM) polymers have limited stability at HTHS conditions (Cui et al., 2016). Modified polyacrylamides such as N-vinyl-pyrrolidone-acrylamide (NVP-AM)/2-acrylamido-2-methyl-1-propane sulfonic acid (AMPS) (Kulawardana et al., 2012; Gaillard et al., 2015; Dupuis et al., 2017b) and SAV polymers are suitable at high temperatures (Panthi et al., 2022). SP floods typically have high surfactant retention because of low pH. Surfactant retention can be reduced by using a salinity gradient and by adding sacrificial agents, e.g., sodium polyacrylate (NaPA) or polyacrylate (ShamsiJazeyi, 2014a; ShamsiJazeyi, 2014b; Koparal et al., 2021; Panthi et al., 2022).

The objective of this work is to develop a surfactant formulation for an off-shore carbonate reservoir at 100°C that has been flooded with seawater during the water flood. Seawater is also the easily available brine for the SP flood in this reservoir. Injecting a salinity gradient during SP flood reduces the surfactant retention, but it is operationally expensive to decrease or increase the salinity of the injection brine in off-shore reservoirs. Another objective of this work is to determine the effect of constant salinity flood (at seawater salinity without a salinity gradient) on oil recovery during the SP flood. Phase behavior tests have been conducted to identify an effective surfactant formulation around seawater salinity. The surfactant components and concentrations were changed to obtain the optimum salinity below and above the seawater salinity. Corefloods were conducted with and without salinity gradients to study the effectiveness of SP floods.

## 2 Methodology

The target reservoir was a limestone reservoir with a light oil at a temperature of 100°C. The porosity (Ø) of the rock was 10%–15%, and the permeability (k) was in the range of 10–60 mD. The oil gravity was approximately 40°API. The oil was not acidic. The dead oil samples were obtained from the field. The salinity of the formation brine was 32,500 ppm.

### 2.1 Chemicals

Both commercially available chemicals and in-house synthesized chemicals were used. Carboxylates were synthesized from alkoxylates provided from Harcros Chemicals. Co-solvents phenol-20EO and IBA-1PO-20EO were also obtained from Harcros Chemicals. 1518IOS and lauryl betaine were obtained from Sasol, and C9-11-8EO alkoxylates were obtained from Shell Chemicals. Polymer SAV 10xv is a terpolymer of acrylamide, acrylamido-tertiary-butyl sulfonate (ATBS), and B-vinyl pyrrolidone (NPV) with a molecular weight 8–10 MD and was provided by SNF Floerger in powder form. Sodium chloride (NaCl), calcium chloride (CaCl_2_), and magnesium chloride (MgCl_2_) were obtained from Fisher Scientific. The injection brine was seawater, and it was prepared with 29,660 ppm NaCl, 4,360 ppm Na_2_SO_4,_ 1,471 ppm CaCl_2_.2H_2_O, and 5,740 ppm MgCl_2_.6H_2_O.

### 2.2 Oil properties

The viscosity of dead oil was 1 cp at the reservoir temperature of 100°C. The measured oil density was 0.78 g/mL, and the molecular weight was 148. Acid number was <0.1 mg KOH/gm, and base number was <0.1 mg KOH/gm. The SARA analysis showed 41.1 wt% saturates, 32.4 wt% aromatics, 26.4 wt% resin, and 0.2 wt% asphaltenes. The crude oil was filtered through a 0.45-micron filter paper under 50 psi at 85°C before using in experiments. The interfacial tension between oil and synthetic seawater was 24 mN/m at 80°C.

### 2.3 Aqueous stability

The aqueous stability experiments were performed for each surfactant mixture prepared for the phase behavior study. Aqueous stability is important for the injection of surfactants into the sand face. The surfactant mixture was solubilized in brine; if there was no precipitation or phase separation, the surfactant was called aqueous stable at that salinity and temperature.

### 2.4 Phase behavior and interfacial tension

Phase behavior experiments were conducted to study the interaction of the surfactant formulation with oil and seawater at the reservoir temperature. Live oil could not be used because these experiments were conducted at ambient pressure and reservoir temperature. Simulated live oil was created by adding 20 wt% toluene to the dead oil based on viscosity and the gas–oil ratio. This oil was used in phase behavior experiments and core floods. Phase behavior tests included careful observation of both the aqueous surfactant mixtures and the mixtures of aqueous surfactant solutions with the simulated live oil over a sufficiently long period of time for them to reach equilibrium or to be rejected as unsuitable for use. Standard 5-mL borosilicate pipettes with 0.1-mL markings were used to create phase behavior scans (with varying salinity); the pipettes were sealed and kept in an oven to equilibrate at the reservoir temperature. Typically, phase behavior scans consist of 10 pipettes with varying salinity, with each pipette being recognized as a data point in the series. The oil amount in the pipette was set to be 30% by volume for most of the experiments, but 10% and 50% were also used in a few experiments (to mimic oil saturations of 30%, 10%, and 50% in reservoirs). Salinity was varied in the range of zero salinity to twice the seawater salinity. Low-salinity samples were prepared by diluting the seawater with deionized water; high-salinity samples were prepared by mixing seawater with twice-seawater salinity water in different proportions. The samples were equilibrated at the reservoir temperature of 100°C, and their phase volumes were observed. The solubilization ratio of oil (or water) in the microemulsion phases was calculated from the phase volumes by dividing the amount of oil (or water) dissolved in the microemulsion phase by the amount of the surfactant. The interfacial tension (IFT) was estimated from the Chun Huh equation (Huh, 1979).

$$\text{IFT}=\frac{0.3}{\sigma^{2}},$$

where σ is the solubilization ratio. When the σ value is 10 or higher, the IFT value would be 0.003 mN/m or less, which is called an ultralow IFT value.

Initial phase behavior screenings identified alkoxy carboxylate surfactants as promising primary surfactants and lauryl betaine and internal olefin sulfonate (IOS) surfactants as co-surfactants in this study.

### 2.5 Surfactant/co-solvent selection

Both high-molecular weight and low-molecular weight alkoxy carboxylate surfactants were tested. Carboxylate surfactants were used as the primary surfactants for the SP formulation due to their stability at high temperature conditions. The surfactants included various numbers of the PO (propylene oxide) and EO (ethylene oxide) groups. By varying the PO and EO in the surfactants, the phase behavior can easily be optimized. For example, if the formulation needed to be more hydrophilic, the number of EOs in the surfactant was increased. The primary surfactants were paired with one or more co-surfactants to improve the phase behavior (increase the solubilization ratio and reduce IFT).

### 2.6 Sand pack preparation

Corefloods were performed in crushed sand packs at the reservoir temperature of 100°C. Reservoir core plugs were not available, but crushed core sand was. The sand was sieved with a mesh of size 150–400 microns. The crushed limestone was packed to form a sand pack that was 1 ft long and 0.82 inches in diameter. This pack mimicked the mineralogy of the reservoir. The sand pack was saturated with seawater by injecting the seawater, and water permeability was measured. The sand pack was then flooded with simulated live oil (20 wt% toluene in dead oil) from the top of the sand pack at a high pressure until no water was present in the effluent. Oil permeability was measured by injection oil at different flow rates, and the pressure drop was recorded. Table 1 describes the properties of the sand pack, oil saturation, and pore volume.

**TABLE 1:** Sandpack properties.

Core flood #	Length (inch)	Diameter (inch)	k (mD)	Φ (%)	Pore volume (mL)	S_oi_ (%)
1	12	0.82	1,620	39	40.76	47
2	12	0.82	517	36.5	45.82	47.1
3	12	0.82	143	39	40.55	40.7
4	12	0.82	202	40.7	42.28	44.7
5	12	0.82	38.28	36.9	38.28	59.8

### 2.7 SP corefloods

Five corefloods were conducted in sand packs to evaluate the oil displacement efficiency of surfactant formulations. After oil saturation, the sand pack was equilibrated at the reservoir temperature before water flood. Water flood was conducted by injecting seawater until no oil was recovered. Water flood was followed by the SP and polymer slugs; the composition of each slug is listed in Table 2. Coreflood #1 was conducted with the surfactant formulation #10; formulation #11 was used for corefloods #2–5. Corefloods #2 and #3 tested different salinity gradients. Corefloods #4 and #5 tested constant salinity at seawater salinity. Coreflood #5 was similar to coreflood #4, except the permeability of the pack was quite low. The polymer concentration in all of the floods was 5,000 ppm SAV 10xv. The SAV 10xv polymer was selected because of its stability at high temperature and high salinity. The polymer concentration was intentionally over-designed to test the efficacy of the surfactant formulations.

**TABLE 2:** Slug compositions in SP flood.

Core flood #	Core	Water flood	SP slug	Polymer slug
1	Reservoir crushed rock	Seawater	40% of seawater 0.25 wt% TDA-35PO-45EO-COO-0.25 wt% 1518IOS 0.75 wt% IBA-1PO-20EO 5,000 ppm SAV 10xv	20% of seawater 5,000 ppm SAV 10xv
2	Reservoir crushed rock	140% of seawater	120% of seawater 0.5 wt% C28-35PO-65EO-COO-0.5 wt% 1518IOS 0.5 wt% lauryl betaine 0.75 wt% IBA-1PO-20EO 5,000 ppm SAV 10xv	20% of seawater 5,000 ppm SAV 10xv
3	Reservoir crushed rock	Seawater	160% of seawater 0.5 wt% C28-35PO-65EO-COO- 0.5 wt% 1518IOS 0.5 wt% lauryl betaine 0.75 wt% IBA-1PO-20EO 5,000 ppm SAV 10xv	Polymer I 40% seawater 5,000 ppm SAV 10xv. Polymer II Seawater 5,000 ppm SAV 10xv
4	Reservoir crushed rock	Seawater	Seawater 0.5 wt% C28-35PO-65EO-COO- 0.5 wt% 1518IOS 0.5 wt% lauryl betaine 0.75 wt% IBA-1PO-20EO 5,000 ppm SAV 10xv	Seawater 5,000 ppm SAV 10xv
5	Reservoir crushed rock	Seawater	Seawater 0.5 wt% C28-35PO-65EO-COO-0.5 wt% 1518IOS 0.5 wt% lauryl betaine 0.75 wt% IBA-1PO-20EO 5,000 ppm SAV 10xv	Seawater 5,000 ppm SAV 10xv

## 3 Results

### 3.1 SP formulation development

Many surfactant formulations were studied, but only fourteen formulations are shown in Table 3. The phase behavior of the oil and brine was studied with several single surfactants, mixtures of two surfactants, mixtures of two surfactants and one co-solvent, and mixture of three surfactants and one co-solvent. The surfactants were selected on the basis of prior knowledge and the nature of the oil. Lauryl betaine was selected because this surfactant is useful for high-temperature reservoirs. The carboxylate surfactant was selected as the primary surfactant in this study as carboxylates perform well, and they survive at high temperature and salinity. C_9-11_-8EO, 1518IOS, and lauryl betaine were selected because these surfactants performed well for similar oils (Panthi et al., 2022). The results of both the aqueous stability and the phase behavior study with the oil are summarized in Table 3.

**TABLE 3:** Phase behavior test summary.

Formuln.#	Surfactant formulation (wt%)	Co-solvent (wt%)	Oil scan (%)	Aqueous stability	Results
1	0.5 wt% lauryl betaine		30	Stable up to 80% SW	Type I
2	0.5 wt% C9-11-8EO		30	Stable up to 50% SW	No interaction
3	0.5 wt% C28-35PO-50EOCOO^-^		30	Stable up to 80% SW	Some interaction with oil
4	0.5 wt% lauryl betaine +0.5% 1518IOS		30	Stable up to 40% SW	Type II
5	0.5 wt% C9-11-8EO + 0.5% 1518IOS		30	Stable up to 40% SW	Type II
6	0.5 wt% C28-35PO-50EOCOO^−^ + 0.5% lauryl betaine		30	Stable up to 220% SW	Type II
7	0.5 wt% lauryl betaine +0.5 wt% C28-35PO-50EO-COO^-^	0.5 wt% Phenol-20EO	30	Stable up to 10% SW	Type II
8	0.5 wt% C_9-11_-8EO+0.4 wt% lauryl betaine +0.4 wt% C_15-18_-IOS		30	Stable up to 120% SW	Type II
9	0.5 wt% TDA-35PO-45EO-COO^-^+ 0.4 wt% 1518IOS		30	Unstable at 10% SW	Type III, but aq. stability is bad
	+0.4 wt% lauryl betaine				
10a	0.25 wt% TDA-35PO-45EO-COO^-^	0.75 wt% IBA-1PO-20EO	30	Seawater stable	Type III, good phase behavior
	+0.25 wt% 1518IOS				
	+0.25 wt% lauryl betaine				
10b	0.25 wt% TDA-35PO-45EO-COO^-^	0.75 wt% IBA-1PO-20EO	50	Seawater stable	Type III, good phase behavior
	+0.25 wt% 1518IOS				
	+0.25 wt% lauryl betaine				
10c	0.25 wt% TDA-35PO-45EO-COO^-^		10	Seawater stable	Type III, good phase behavior
	+0.25 wt% 1518IOS				
	+0.25 wt% lauryl betaine				
11	0.5 wt% C28-35PO-50EO-COO^-^	0.75 wt% IBA-1PO-20EO	30	Seawater stable	Type III, good phase behavior
	+0.5 wt% 1518IOS				
	+0.5 wt% lauryl betaine				
12	0.6 wt% C28-35PO-50EO-COO^-^	0.9 wt% IBA-1PO-20EO	30	Seawater stable	Type III, good phase behavior
	+0.6 wt% 1518IOS				
	+0.6 wt% Lauryl betaine				

In the first three phase behavior experiments (1–3), single surfactants were used, but none of them showed good oil solubilization. After single-surfactant experiments, mixtures of two surfactants were used in the study (formulations 4–6). As the mixture of two surfactants also did not produce good phase behavior, a co-solvent was added to the mixture of two surfactants, as shown in formulation #7. The mixture of two surfactants and one co-solvent also did not produce good phase behavior. In phase behavior formulation 8, a mixture of three surfactants (0.5% C_9-11_-8EO + 0.4% lauryl betaine +0.4% C_15-18_-IOS) was used, but only Windsor type-II phase behavior was observed. Aqueous stability of the phase behavior formulations was also studied in parallel. The aqueous stability of formulations 1–7 was not good at seawater salinity, but the aqueous stability of formulation #8 was good. In formulation #9, the alkoxylate surfactant was replaced with the carboxylate, and it produced good Windsor type-III phase behavior, but the aqueous stability was not good. In formulation #10, the concentration of each surfactant was reduced from those of formulation #9, and it increased the aqueous stability with a better phase behavior (described next).

#### 3.1.1 Phase behavior formulation #10a (30% oil scan)

**FIGURE 1:**
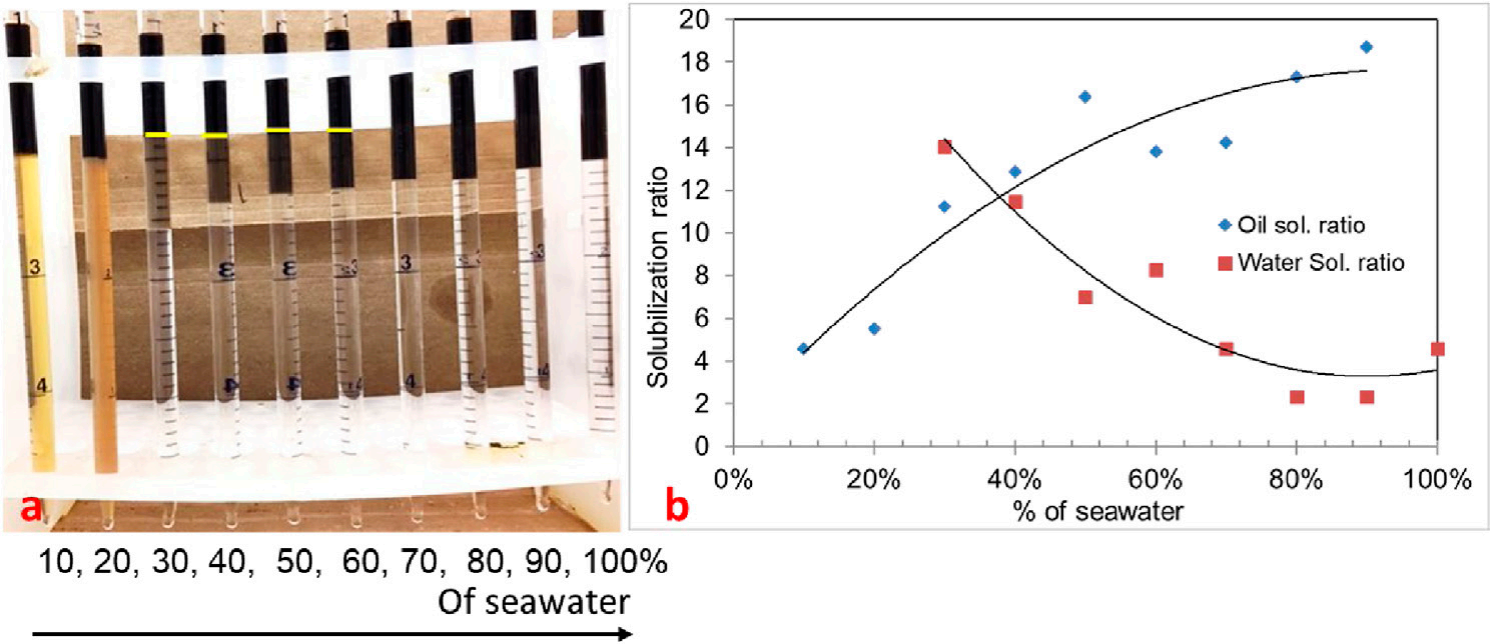
**(A)** Phase behavior of 0.25 wt% TDA-35PO-45EO-COO^-^ + 0.25 wt% lauryl betaine +0.25 wt% C_15-18_-IOS +0.75 wt% IBA-1PO-20EO (30% oil). **(B)** Solubilization ratio plot.

In the phase behavior formulation #10a, a mixture of surfactants 0.25 wt% TDA-35PO-45EO-COO^-^, 0.25 wt% lauryl betaine, and 0.25 wt% C_15-18_-IOS and 0.75 wt% IBA-1PO-20EO co-solvent was considered, and a salinity scan was conducted with 30% oil (70% aqueous solution). In this experiment, all Windsor types (I, II, and III) of phase behavior were observed, but the optimum salinity was low. The salinity region from 30% to 60% (of the seawater salinity) showed type-III (ultralow) phase behavior, as shown in Figure 1A. The solubilization ratio of this phase behavior is shown in Figure 1B. The optimum salinity was at 38% of the seawater salinity. The interfacial tension was calculated to be 0.002 mN/m by using the Chun Huh equation.

#### 3.1.2 Phase behavior formulation #10b (50% oil scan)

**FIGURE 2:**
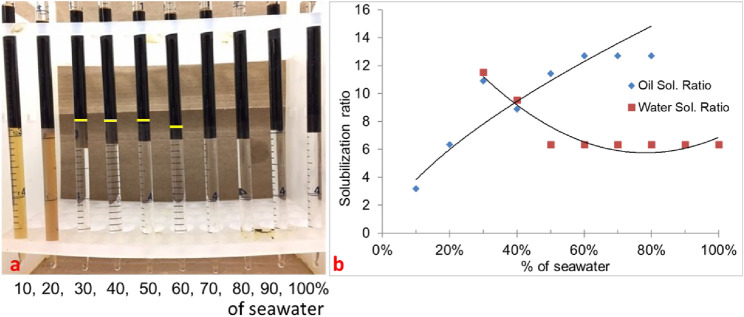
**(A)** Phase behavior of 0.25 wt% TDA-35PO-45EO-COO^-^ + 0.25 wt% lauryl betaine +0.25 wt% C_15-18_-IOS +0.75 wt% IBA-1PO-20EO (50% oil scan). **(B)** Solubilization ratio plot.

In formulation #10b, 50% oil (water-to-oil ratio = 1) was used with the same surfactant system, and the results are shown in Figure 2. The Windsor type-III region is similar to that of 30% oil scan, and there is a minor shift in optimum salinity. This is because the oil is not active and no alkali is used. The optimum salinity was obtained at approximately 40% of the seawater salinity. The IFT was obtained to be 0.0034 mN/m.

#### 3.1.3 Phase behavior formulation #10c (10% oil scan)

**FIGURE 3:**
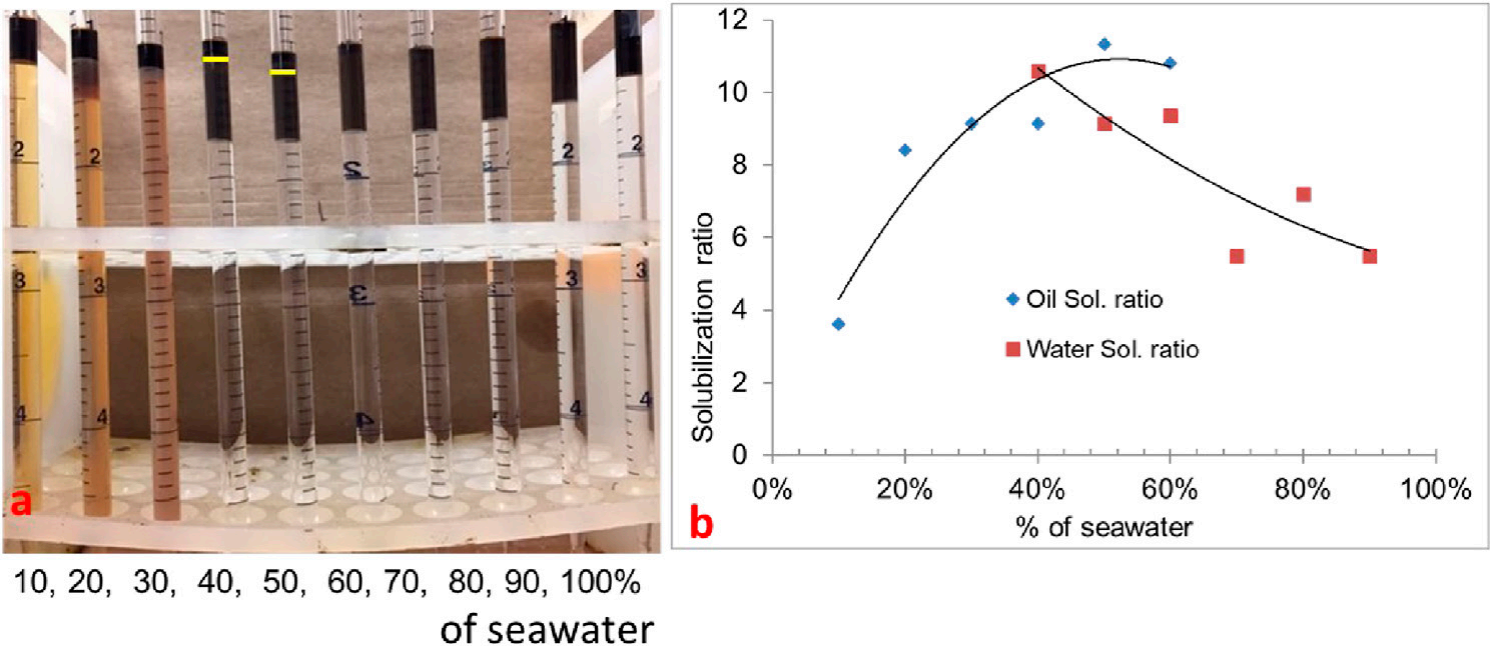
**(A)** 0.25 wt% TDA-35PO-45EO-COO^-^ + 0.25 wt% lauryl betaine +0.25 wt% C_15-18_-IOS +0.75 wt% IBA-1PO-20EO (10% oil scan). **(B)** Solubilization ratio plot.

In formulation #10c, 10% oil (water–oil ratio = 9) was used with the same formulation. In this scan, the oil solubilization ratio was high, but it was difficult to quantify because the microemulsion phase was quite dark, as shown in Figure 3A. The solubilization ratio is shown in Figure 3B. The optimum salinity was approximately 40% of seawater salinity, and IFT was estimated to be 0.0027 mN/m.

#### 3.1.4 Phase behavior formulation #11

**FIGURE 4:**
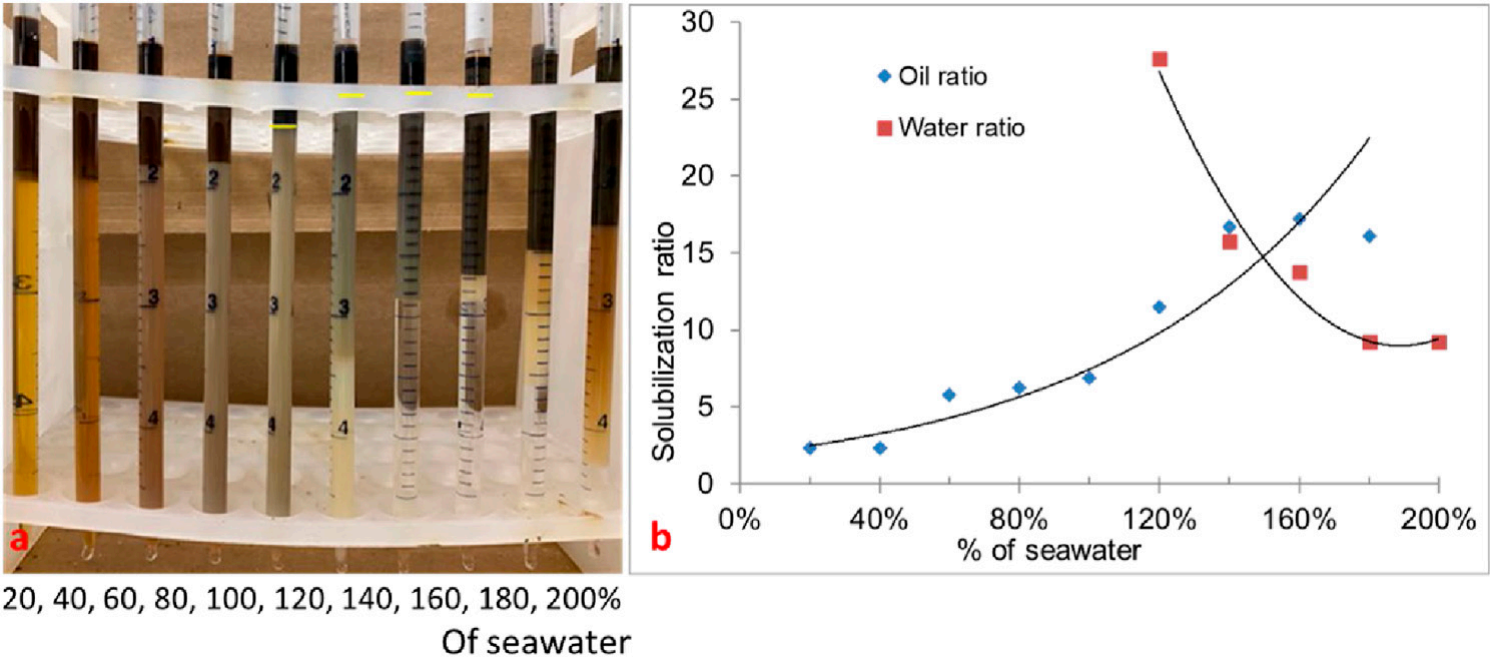
**(A)** Phase behavior of formulation #11 containing 0.5 wt% C28-35PO-50EO-COO^-^ + 0.5 wt% lauryl betaine +0.5 wt% IOS +0.75 wt% IBA-PO-EO (30% oil). **(B)** Solubilization ratio plot.

Though formulation #10 showed low tension phase behavior, the optimal salinity was low. The goal of this study was to have an optimum salinity close to seawater salinity. In formulation #11, the phase behavior of a mixture of surfactants (0.5 wt% C28-35PO-50EO-COO^-^, 0.5 wt% lauryl betaine, and 0.5 wt% C_15-18_-IOS) and 0.75 wt% IBA-1PO-20EO co-solvent was studied. In this experiment, the oil amount was 30%, and all types (I, II, and III) of phase behavior were observed. The salinity region from 120% to 160% (seawater salinity) showed type-III (ultralow IFT) phase behavior, as shown in Figure 4A. Figure 4B shows the solubilization ratio plot. The optimum salinity was obtained at approximately 150% of the seawater salinity, with a solubilization ratio of 15. The interfacial tension calculated by the Chun Huh equation was 0.0015 mN/m. The three-phase region in this formulation came close to the seawater salinity. This formulation was tested in corefloods.

#### 3.1.5 Phase behavior formulation #12

**FIGURE 5:**
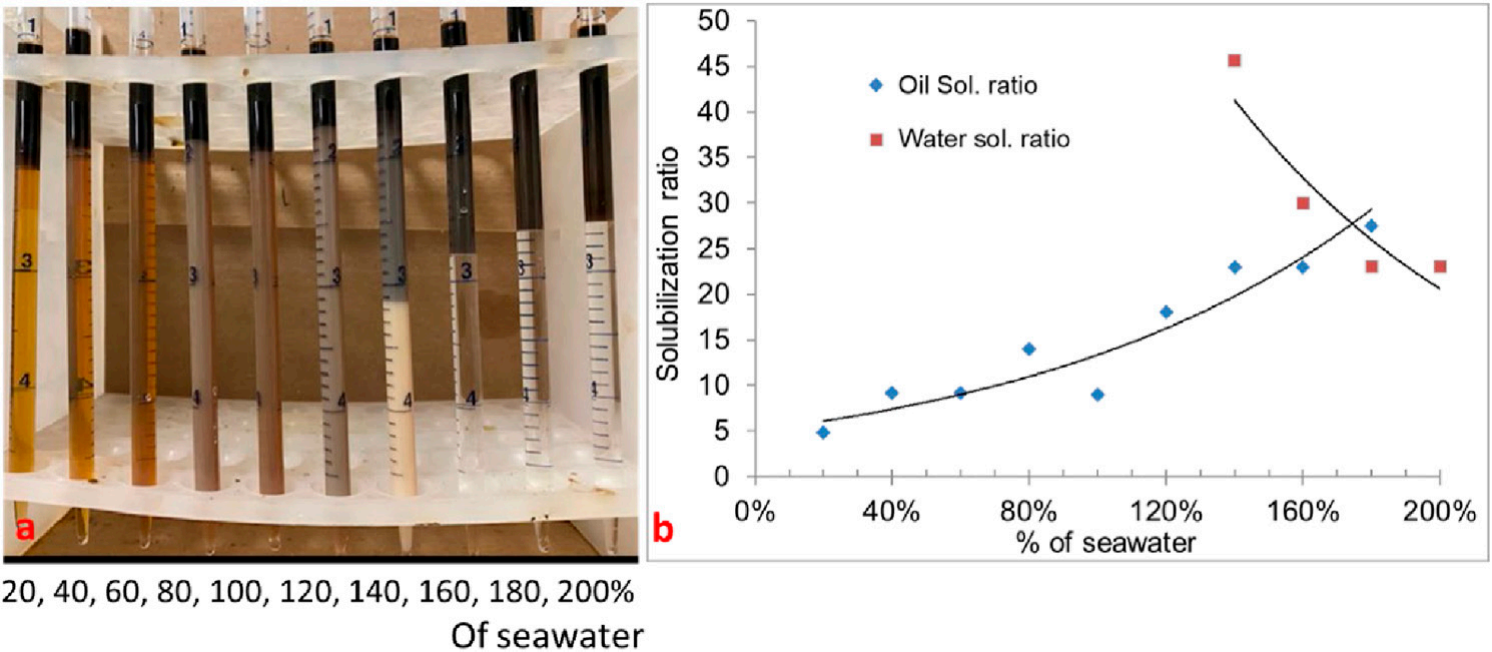
**(A)** Phase behavior of 0.6 wt% carboxylate +0.6 wt% lauryl betaine +0.6 wt% IOS +0.9 wt% IBA-1PO-20EO) with diluted/concentrated reservoir seawater (30% oil scan). **(B)** Solubilization ratio plot.

Formulation #12 consisted of the same surfactants as in formulation #11, but at a higher concentration (0.6 wt% C28-35PO-50EO-COO^-^, 0.6 wt% lauryl betaine, and 0.6 wt% C_15-18_-IOS) and 0.75 wt% IBA-1PO-20EO co-solvent. The phase behavior pipettes are shown in Figure 5A. The result is promising (displaying all the Windsor types with a high solubilization ratio). The salinity region from 140% to 180% seawater salinity showed type-III (ultralow) phase behavior. Figure 5B shows the solubilization ratio for the 30% oil scan. The optimum salinity was obtained at approximately 170% of the seawater salinity, with a solubilization ratio of 27. The interfacial tension was calculated to be 0.0004 mN/m, which is ultralow. The optimum salinity was much higher than desired. These phase behavior experiments showed that the choice of surfactants and the concentrations affect the optimal salinity and can be moved from below to above seawater salinity.

### 3.2 SP flood in crushed rock packs

Five surfactant–polymer floods were conducted in reservoir crushed rock sand packs. All the floods used the simulated live oil. The SP slug compositions of all the floods are presented in Table 2. Formulation #10 was tested in flood 1, while formulation #11 was tested in floods 2–5. The polymer concentration in all of the floods was 5,000 ppm SAV 10xv. Seawater was used in the water flood in all the floods, except flood 2. In flood 2, 140% of seawater salinity was used for water flood. In floods 1, 2, and 3, the salinity gradient was used; SP and polymer slugs were at different salinities. In floods 4 and 5, only seawater was used for all the slugs (constant salinity seawater floods). The viscosity of SP and polymer slugs was measured with a DHR-3 rheometer. Table 1 shows the initial conditions for the corefloods, and their results are summarized in Table 4.

**TABLE 4:** Oil recovery in sand pack flood experiments.

Core flood#	Formulation #	Salinity gradient	Soi (%)	WF oil recovery (%OOIP)	WF + ASP recovery (%OOIP)	ASP recovery (%OOIP)	Residual oil sat. (%)
1	10	Yes	47	44	68	24	15
2	11	Yes	47.1	51	79	28	9.7
3	11	Yes	40.7	66	>99	33	<0.5
4	11	No	44.7	67.3	98.1	0.8	1
5	11	No	59.8	48	91	43	5

#### 3.2.1 SP flood 1 (surfactant formulation 10)

The SP formulation #10 was considered for this coreflood test. The SP flood in the sand pack was conducted in a vertical mode (to conduct stable flood) where the injectants were injected from the bottom and the effluent was collected from the top of the core. The sand pack flood was performed at the reservoir temperature of 100°C. First, the sand pack was saturated with seawater, and then the seawater was displaced with oil to reach the initial oil saturation, S_oi_. The properties of the sand packs are listed in Table 1. The pore volume of the sand pack was 40.76 mL, and the water permeability was obtained to be 1.62 Darcy. Then, the sand pack was flooded with 2 PV of seawater to represent the water flood, which was followed by 0.5 PV of SP slug injection. The SP slug was composed of 40% seawater salinity water, 0.25 wt% TDA-35PO-45EO-COO^-^, 0.25 wt% lauryl betaine, 0.25 wt% C15-18-IOS, 0.75 wt% IBA-1PO-20EO, and 5,000 ppm polymer (SAV10xv). After SP injection, the core was flooded with 2 PV of the polymer chase slug. The polymer chase was composed of 20% seawater salinity water and 5,000 ppm polymer.

**FIGURE 6:**
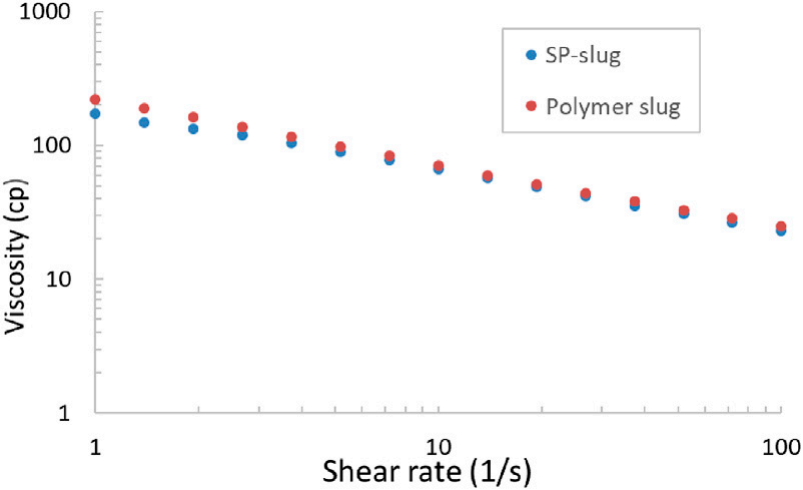
Viscosity measurement of SP and polymer slugs at room temperature.

The viscosities of both SP slug and polymer chase were measured in a rheometer at 25°C, and the result is shown in Figure 6. The viscosity at 10 s^-1^ is similar for both SP slug and polymer chase; it is approximately 70 cp at 25°C. The viscosity is lower at 100°C. The viscosity at the reservoir temperature was not measured, but it can be estimated.

**FIGURE 7:**
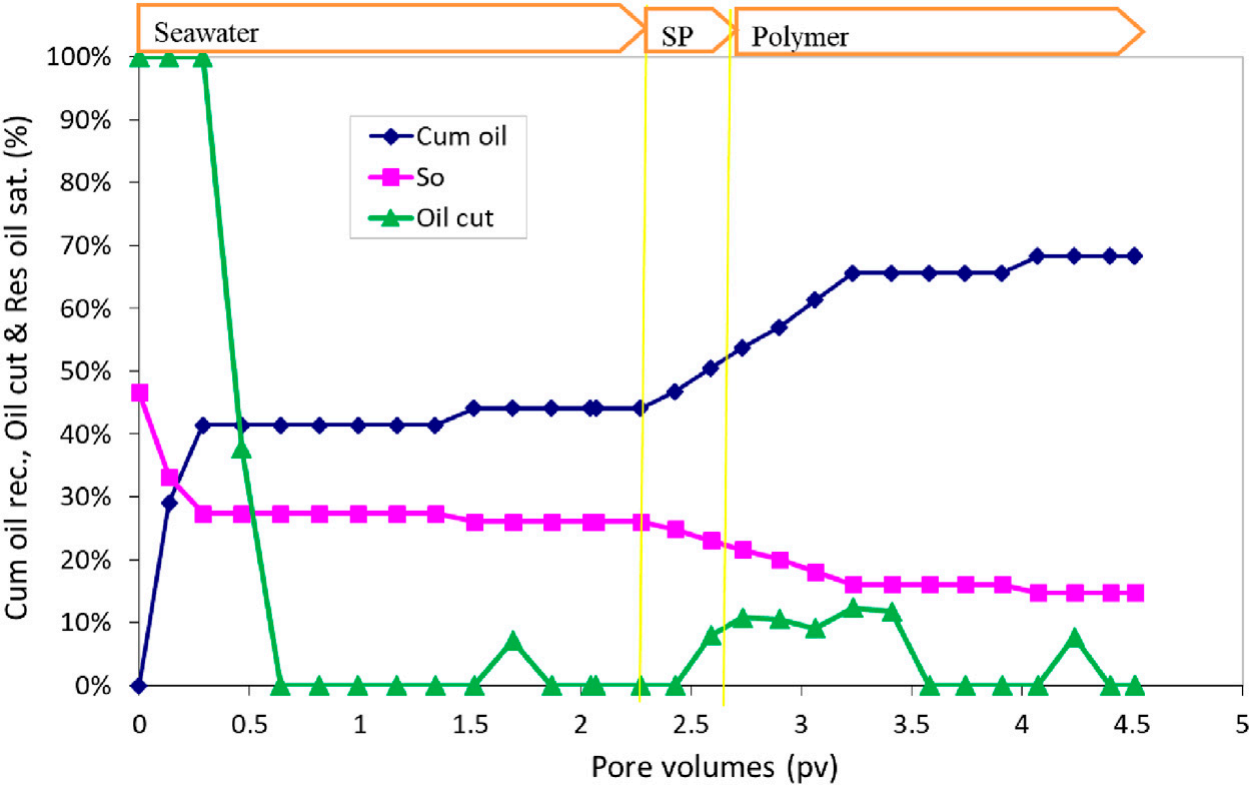
Oil recovery, oil cut, oil saturation, and pressure drop of SP flood 1.

The oil recovery, oil cut, and residual oil saturation are presented in Figure 7. The initial oil saturation was 47%. Water flood at 0.028 mL/min (1 ft/day) injection rate recovered approximately 44% OOIP, and the oil saturation was reduced to 26%. After water flood, 0.5 PV of the SP slug was injected at the same flow rate 0.028 mL/min (1 ft/day). The SP slug was followed by 2 PV of chase polymer injection at the same flow rate. The oil recovery increased to 68% OOIP. The residual oil saturation at the end of chemical flood was 15%. The effluent oil cut increased to only 12%, but oil kept coming out at a low oil cut during the first PV injection of the polymer. The pressure drop could not be presented, but the maximum pressure obtained was approximately 5 psi during the SP and polymer injection. The effluent surfactant concentration was measured with high-performance liquid chromatography (HPLC). The retention of the surfactant in the sand pack was calculated from the effluent surfactant concentrations by using a material balance. The retention was 0.183 mg per gm of rock. The oil recovery was low due to the quality of surfactant formulation, the low surfactant concentration (0.75 wt%), and adsorption.

#### 3.2.2 SP flood 2 (surfactant formulation 11)

The SP formulation #11 was tested in the sand pack flood 2. The pore volume of the sand pack was obtained to be 45.82 mL, and the water permeability was obtained to be 517 mD. After oil saturation, water flood was conducted with a brine of 140% seawater salinity (1.7 PV). The water flood was followed by 0.5 SP slug injection at a flow rate of 0.032 mL/min (1 ft/day). The SP slug was composed of 120% seawater salinity water, 0.5 wt% C28-35PO-50EO-COO^-^, 0.5 wt% lauryl betaine, 0.5 wt% C15-18IOS, 0.75% IBA-1PO-20EO, and 5,000 ppm polymer (SAV10xv). After SP injection, the sand pack was flooded with approximately 2.2 PV of the polymer solution. The polymer chase solution was composed of 20% salinity water with 5,000 ppm polymer.

**FIGURE 8:**
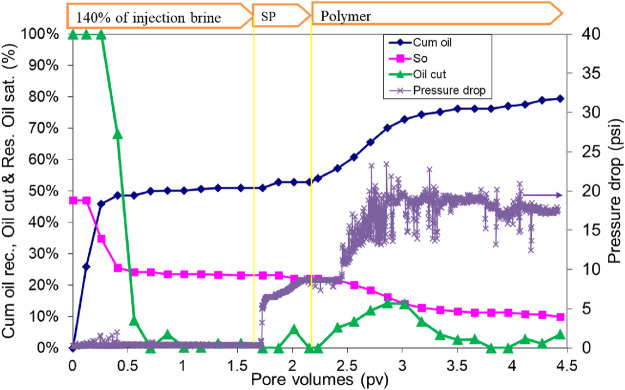
Oil recovery, oil cut, oil saturation, and pressure drop during SP flood 2.

The oil recovery, oil cut, and residual oil saturation are presented in Figure 8. The initial oil saturation was 47.1%. Water flood recovered approximately 51% OOIP. The cumulative oil recovery for the SP flood was 79% OOIP. The salinity in the polymer slug was only 20% of the seawater. The effluent oil cut increased to approximately 15% during the polymer injection. The residual oil saturation at the end of the SP flood was 9.7%. The pressure drop during the water flood was approximately 0.3 psi; it increased to approximately 7 psi during the SP flood, and during polymer injection, the pressure drop further increased to approximately 18 psi and eventually decreased to approximately 16 psi at the end of the flood.

**FIGURE 9:**
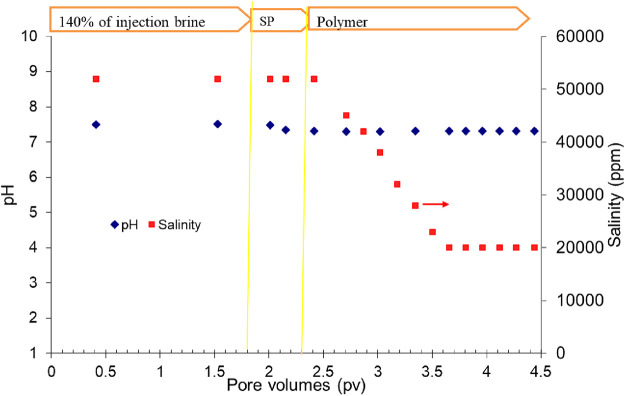
Salinity of the effluent samples in SP flood 2.

The pH and salinity of the effluent samples collected during the flood were measured and are shown in Figure 9. The pH was almost constant throughout the experiment. At the beginning, the pH was approximately 7.5 and decreased to approximately 7.3 during SP and polymer flood. The salinity of the effluents at the beginning of the flood was approximately 52,000 ppm. The salinity started to decrease at the beginning of the polymer flood and decreased to 20,000 ppm toward the end of the flood. It is because the water flood had the salinity of approximately 52,000 ppm that the SP slug had the salinity of approximately 48,000 ppm and the polymer slug had the salinity of approximately 8,000 ppm.

**FIGURE 10:**
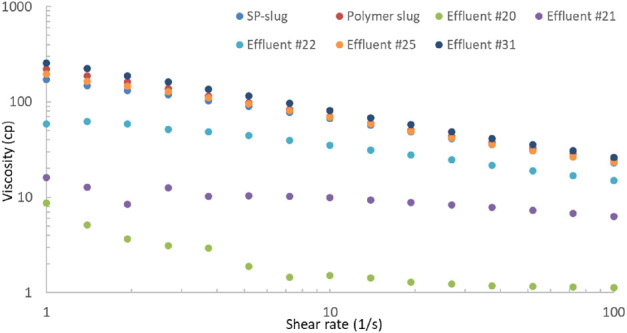
Viscosity of effluent water samples in SP flood 2.

The viscosity of effluent samples was measured and is shown in Figure 10. The viscosity of effluent sample #20 was low and almost like water viscosity. The surfactant and polymer had not broken through yet. The viscosity increased for the effluent samples #21 and #22. The rest of the effluents were viscous until the end of the flood. Effluent #31 is the last one during the coreflood. The viscosity of SP and polymer slugs is also presented in this plot. The viscosities of the later effluents were similar or slightly higher than that of the injection slugs because of evaporation of water in these effluent samples. The oil recovery increased due to SP slug, but it was still under 90% recovery, possibly due to a high salinity gradient in the flood.

#### 3.2.3 SP flood 3 (surfactant formulation 11 with redesign of salinity gradient)

The SP formulation #11 used for last flood was re-tested in a sand pack flood with some improvement in salinity gradient and polymer slug design. The pore volume of the sand pack was obtained to be 40.55 mL, and the water permeability was obtained to be 143 mD. After oil saturation, the sand pack was heated at a reservoir temperature for few hours, and water flood was conducted with seawater. The water flood was followed by 0.5 PV SP slug injected at a flow rate of 2 ft/day. The SP slug was composed of 160% seawater salinity water, 0.5 wt% C28-35PO-50EO-COO^-^, 0.5 wt% lauryl betaine, 0.5 wt% C_15-18_IOS, 0.75% IBA-1PO-20EO, and 5,000 ppm polymer (SAV10xv). After SP injection, the sand pack was flooded with two different polymer slugs (polymer I and polymer II slugs). SP flood was followed by 0.5 PV of polymer I slug, which is composed of 40% seawater salinity water with 5,000 ppm polymer. This reduction in salinity leads to adsorption reduction and mobilization of the surfactant bank. Polymer I was further followed by 2.3 PV of the polymer II slug, which consists of seawater in the presence of 5,000 ppm polymer. This salinity is convenient in the field, and most of the polymer injections were conducted at this salinity.

**FIGURE 11:**
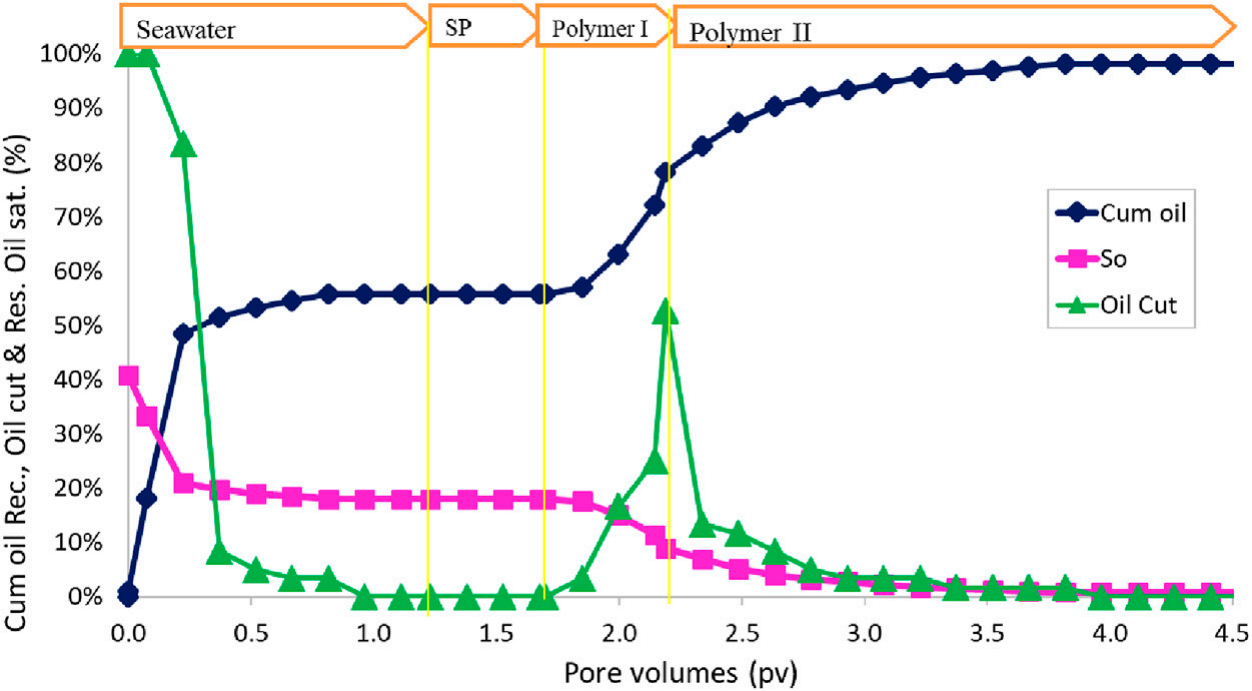
Oil recovery, oil cut, oil saturation, and pressure drop during SP flood 3.

The oil recovery, oil cut, and residual oil saturation are presented in Figure 11. The initial oil saturation was 40.7%. Water flood at 0.057 mL/min (2 ft/day) injection rate recovered approximately 66.0% OOIP. The cumulative oil recovery at the end of polymer II injection increased to 99.9% OOIP. The salinity in the polymer I slug was only 40% of the seawater, but in the polymer II slug, the salinity was seawater salinity. The effluent oil cut increased to approximately 30% for a short time during the polymer injection. The residual oil saturation at the end of the polymer II flood was very small, approximately 0.1%. The oil recovery was high in this flood due to the correct salinity gradient and an effective surfactant formulation.

After the SP flood was over, the sand was taken out to see if there was some oil left. The sand was clear without any visible oil. Approximately 500 mL of water was mixed with the sand and stirred to segregate oil and allow it to collect at the top of the water, but we could not see any oil droplets suspended in the water. This confirms that almost all the oil was recovered during the SP flood.

This flood and flood 2 have the same surfactant formulation #11. This flood has the recovery of approximately 99.9% OOIP, but flood 2 has the recovery of 79% OOIP. The reason for the low recovery in flood 2 was the incorrect salinity gradient. The optimum salinity used was 140% seawater. We did water flood with this salinity, and SP flood was conducted with 120% seawater and polymer flood with 20% seawater. This salinity gradient might have shortened the type III region. Flood 3 shows that the surfactant formulation #11 is effective with a proper salinity gradient. In the next two floods, formulation #11 will be tested with no salinity gradient and a constant salinity of seawater salinity.

#### 3.2.4 SP flood 4 (surfactant formulation 11 with constant seawater salinity)

As the formulation 11 recovered all of the oil with the correct salinity gradient (flood 3), flood 4 tested the same chemicals at a constant seawater salinity. The water flood, SP slug, and the polymer drive used only seawater. The pore volume of the sand pack was 42.3 mL, and the water permeability was 202 mD. After oil saturation, the sand pack was heated at a reservoir temperature for few hours, and water flood was conducted with seawater. The water flood was followed by a 0.5 PV SP slug injection at a flow rate of 0.5 ft/day. The SP slug (formulation #11) was composed of seawater salinity, 0.5 wt% C28-35PO-50EO-COO^-^, 0.5 wt% lauryl betaine, 0.5 wt% C15-18IOS, 0.75% IBA-1PO-20EO, and 5,000 ppm polymer (SAV 10xv). SP flood was further followed by 1.5 PV of polymer slug, which is composed of seawater salinity with 5,000 ppm polymer.

**FIGURE 12:**
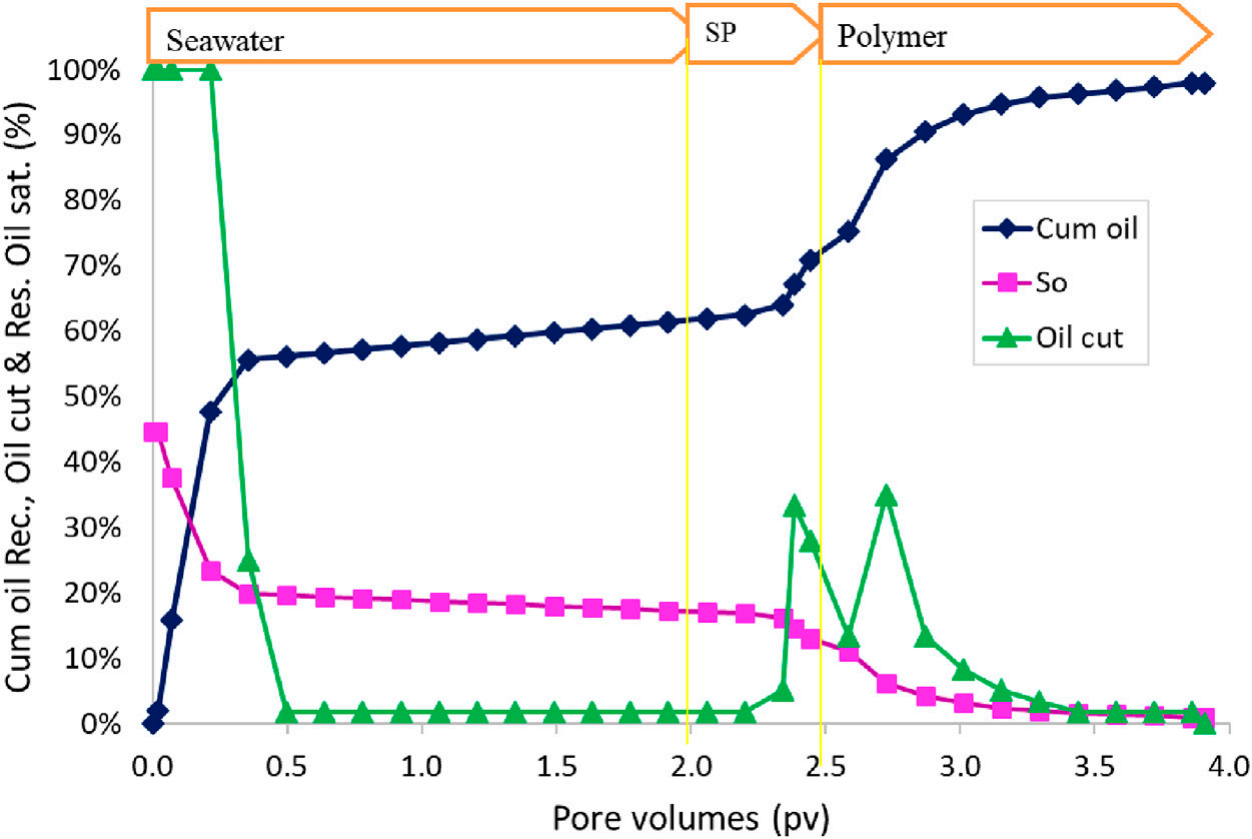
Oil recovery, oil cut, and oil saturation during SP flood 4.

The oil recovery, oil cut, and residual oil saturation are presented in Figure 12. The initial oil saturation was 44.7%. Water flood at 0.057 mL/min (2 ft/day) injection rate recovered approximately 60.5% OOIP. After waterflood, 0.5 PV of the SP slug was the injection rate of 0.014 mL/min (1/2 feet/day. The SP slug was followed by 1.5 PV of polymer injection at 0.028 mL/min (1 ft/day) injection rate. The cumulative oil recovery at the end of the polymer injection was increased to 98.1% OOIP. Oil recovery at 1 PV chemical injection was 92% OOIP. Both slugs were at seawater salinity. The effluent oil cut increased to approximately 30% for a short time during the polymer injection. The residual oil saturation at the end of the polymer flood was 1%. To further quantify the remaining oil, the pack was dismantled, and toluene was added to extract any oil. It showed the presence of only 0.35 mL of oil with UV–Vis absorption spectrometry, consistent with an oil saturation of 1%. The oil recovery was high in this flood, even though the salinity was constant at the seawater salinity and slightly below the optimal salinity.

#### 3.2.5 SP flood 5 (surfactant formulation 11 in low-permeability sand pack)

The SP formulation used for flood #4 was re-tested in flood #5, again without any salinity gradient, but at a low permeability of 38 mD. Oil was injected to achieve an initial oil saturation of 59.8% at room temperature. After oil saturation, the sand pack was heated to the reservoir temperature. Then, the water flood was conducted with the seawater first at 2 ft/day. The water flood was followed by a 0.5 PV SP slug injection at a flow rate of 0.5 ft/day. The SP slug included seawater, 0.5 wt% C28-35PO-50EO-COO^-^, 0.5 wt% lauryl betaine, 0.5 wt% C15-18IOS, 0.75% IBA-1PO-20EO, and 5,000 ppm polymer (SAV 10xv). SP flood was further followed by 2.3 PV of polymer slug, which was composed of seawater and 5,000 ppm polymer.

**FIGURE 13:**
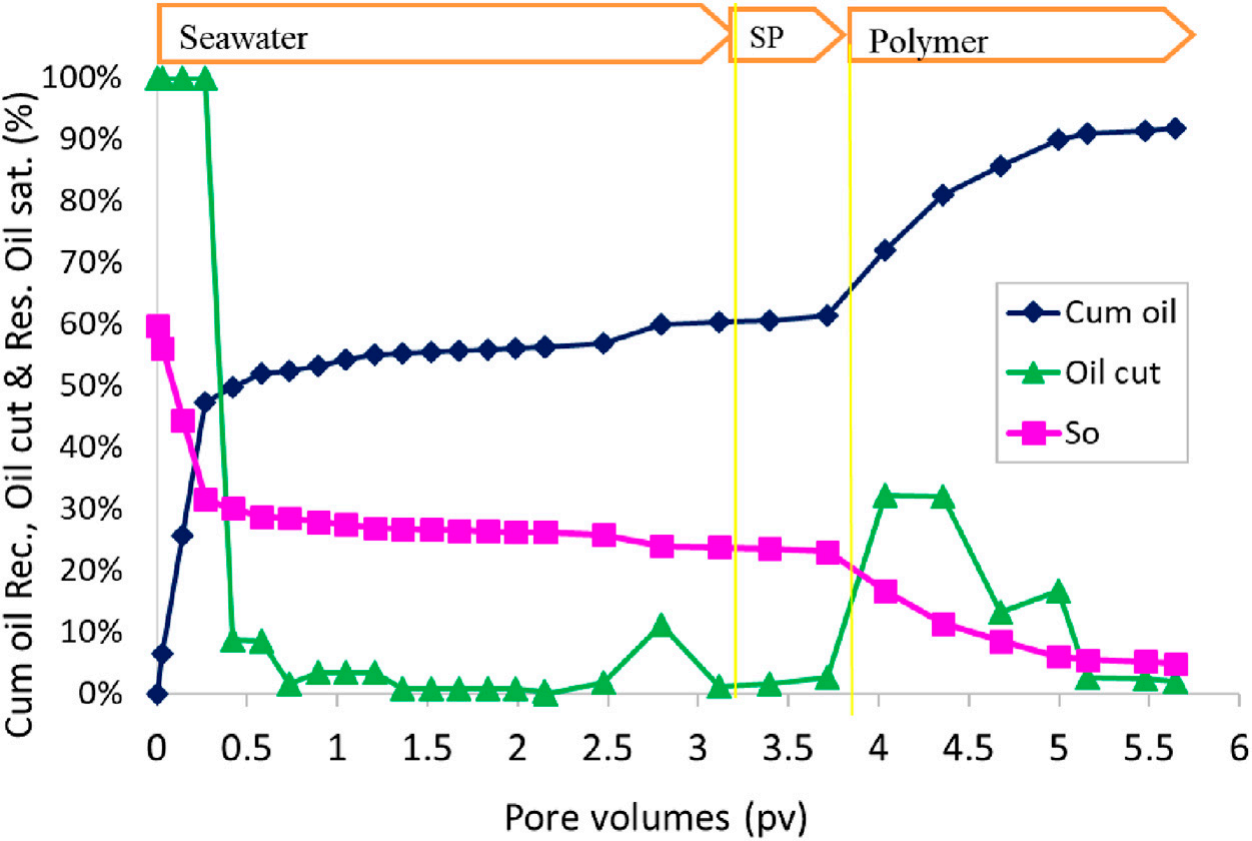
Oil recovery and oil cut as a function of injected pore volume during SP flood 5.

At the end of the water flood, the oil recovery was 60% OOIP. Oil cut started to increase at 0.35 PV slug injection (Figure 13). Oil cut was maintained at approximately 30% at the beginning of polymer injection. The final oil recovery for constant-salinity SP flood was 91% OOIP in the 38.6 mD sand pack. The residual oil saturation was 5%. The oil recovery at 1 PV of the polymer was approximately 88% OOIP. The oil recovery is slightly lower than that tested in a 202 mD sand pack (98%) with a constant-salinity flood.

## 4 Discussion

The injection water is typically seawater for water flood for off-shore reservoirs. However, seawater has a significant amount of divalent cations, Mg^2+^ and Ca^2+^. Softening the seawater is feasible, but it is challenging in the off-shore environment. In this work, we have developed SP formulations compatible with hard brines so that the seawater does not need to be softened. Again, it is not easy to implement a salinity gradient in off-shore conditions; a vast amount of fresh water has to be shipped to the injection site. The injection of seawater (and a constant salinity SP flood) is the easiest. The uniqueness of this research work is the development of ultralow IFT surfactant formulations with seawater for a high-temperature (100°C) carbonate reservoir. The combination of carboxylate, betaine, and internal olefin sulfonate surfactants can withstand the hardness of the seawater and give the ultralow tension and aqueous stability.

This study shows that the SP formulation recovers almost all oil from sand packs of 200–500 mD with a salinity gradient. The formulation with constant-salinity seawater also recovers more than 90% OOIP. Therefore, our research study showed that constant seawater salinity SP floods with seawater can enhance oil recovery significantly if the optimum salinity is not far from the seawater salinity. The operators can evaluate the two different processes (salinity gradient and constant salinity) economically and determine the correct design. Constant seawater SP flood would be easier to conduct. The salinity gradient SP flood can be conducted with the mixing of deionized water and additional salts with the seawater. The higher oil recovery should be weighed against the procurement and mixing of deionized water and salts in the off-shore environment. This process can be considered for many off-shore fields that have been water-flooded with seawater.

## 5 Conclusion

Ultra-low IFT surfactant formulations were developed for an off-shore carbonate reservoir at a high temperature in this research work. Off-shore reservoirs are often water-flooded with seawater. Five SP corefloods were conducted to demonstrate the effectiveness of the formulations. The following conclusions can be drawn from this study:

• Three surfactants were required to produce ultralow IFT behavior with the reservoir oil in the presence of seawater and its dilutions.
• Three promising phase behavior formulations (10, 11, and 12) were identified with optimal salinities close to the seawater salinity. The formulations consisted of carboxylate, lauryl betaine, and internal olefin sulfonate surfactants in the presence of a co-solvent.
• SP core flood with a proper salinity gradient recovered 99.9% OOIP and led to a residual oil saturation of 0.1%.
• Constant seawater salinity SP floods recovered at least 91% OOIP, even for the low-permeability sand pack of 38 mD.
• Constant-salinity SP floods with seawater enhanced oil recovery significantly, even though the recovery is lower than that of the proper-salinity gradient SP flood.
• The surfactant retention was low (0.183 mg/g of rock).

## Figures and Tables

**Table:** 

**ASP**	Alkaline surfactant polymer
**CaCl**_**2**_	Calcium chloride
**DI**	Deionized
**EOR**	Enhanced oil recovery
**ft/day**	Feet per day
**HTHS**	High temperature high salinity
**HPAM**	Hydrolyzed polyacrylamide
**IFT**	Interfacial tension
**S**_**oi**_	Initial oil saturation
**MgCl**_**2**_	Magnesium chloride
**mD**	Millidarcy
**mM**	Millimolar
**NaPA**	Sodium polyacrylate
**OOIP**	Original oil in place
**KCl**	Potassium chloride
**KOH**	Potassium hydroxide
**Ø**	Porosity
**K**	Permeability
**PV**	Pore volume
**PO**	Propylene oxide
**NaCl**	Sodium chloride
**SP**	Surfactant polymer
**WOR**	Water-to-oil ratio

## Data Availability

The original contributions presented in the study are included in the article/Supplementary Material; further inquiries can be directed to the corresponding author.
